# Will Africa’s future epidemic ride on forgotten lessons from the Ebola epidemic?

**DOI:** 10.1186/s12916-015-0359-7

**Published:** 2015-05-14

**Authors:** Oyewale Tomori

**Affiliations:** Nigerian Academy of Science, Academy House, 8A Ransome Kuti Road, University of Lagos, Akoka, Yaba Lagos State Nigeria, University of Lagos Post Office, PMB 1004 Akoka Yaba, Lagos State Nigeria

**Keywords:** Aid dependency, Capacity, Collaboration, Communications, Disease surveillance, Ebola virus disease, International assistance, Lessons learned, Preparedness, Priorities

## Abstract

The Ebola virus disease (EVD) outbreak in the three West Africa countries of Guinea, Liberia, and Sierra Leone, was declared by the World Health Organization (WHO) as a public health event of international concern in August 2014. The disease, which has caused more than 10,000 deaths from over 25,000 cases, has thrived on a failed disease control system, national denial, and a poor and fragile healthcare delivery system. The slow and initially uncoordinated national and global response turned the outbreak into an unprecedented humanitarian disaster. Prevention and control of future outbreaks depend on improving and upgrading disease surveillance into a responsive component of a reliable and efficient health care delivery system. Appropriate capacity building with a conducive operating environment, which has been lacking in the past few decades, will be key to the health system strengthening.

## Background

The World Health Organization (WHO) declared the 2014 Ebola virus disease (EVD) outbreak a public health event of international concern 8 months after the index case occurred and 5 months after it was officially reported [1]. Three countries (Guinea, Liberia, and Sierra Leone) bore the greatest burden of the epidemic, while three other West African countries (Mali, Nigeria, and Senegal) successfully contained the importation of the disease. In addition to the outbreaks in West African countries, the disease was also imported to the USA, UK, and Spain [2]. During the same period, another EVD outbreak unrelated to the West African outbreaks and which was successfully contained occurred in the Democratic Republic of the Congo (DRC) [3]. As of April 15, 2015, the reported number of cases associated with the West African epidemic, which has gone on for the last 16 months, is 25,826 cases with 10,704 deaths [4].

This commentary focuses on issues, generally not publicly discussed, but which have perennially hampered the ability of many African countries to effectively control disease outbreaks. Addressing these issues, and the lessons learned from the ongoing EVD epidemic, will equip African countries to prevent a repeat of the current humanitarian disaster.

## The index case for the current epidemic

The index case of the current West African EVD outbreak is considered to be a 2-year-old boy infected in Meliandou, near Gueckedou, in Guinea, in December 2013 [5]. The WHO was notified late in March 2014, by which time, Guinea had recorded 49 cases and 29 deaths [6]. On August 8, 2014, with a total of 1,779 cases and 961 deaths in Guinea, Liberia, Sierra Leone, and Nigeria [1], the WHO declared the EVD outbreak a public health event of international concern, that is, an ‘extraordinary event’ and a public health risk to other States [1].

## The Nigerian Ebola virus disease (EVD) outbreak

The Nigerian index case of EVD was an individual who had visited and cared for a sibling who died from the disease in Liberia [7,8]. He travelled to Lagos, Nigeria, on a commercial flight, arriving on July 20, 2014, by which time he was acutely ill, and was admitted to a private hospital with fever, vomiting, and diarrhea [7,8]. He was initially treated for malaria having denied any exposure to EVD. His failure to respond to malaria treatment and his arrival from an EVD-affected country led to a suspicion of a case of EVD. Subsequent laboratory tests confirmed the diagnosis, and local public health authorities were alerted of the case of EVD [7,8]. The patient died on July 25, 2014.

The limited spread of EVD in Nigeria was a combination of fortuitous circumstances and the institution of a rapid and aggressive public health and infection control measures. The index case, a diplomat, was sick on arrival in Lagos, at a time when doctors were on strike and government hospitals closed. He was subsequently admitted to a private hospital, where he was the source of infection for nine of the hospital workers, and who were then sources of infection for five others [7,8]. This is in addition to two of the protocol officers who attended to him at the airport. One of those EVD infected protocol officers later escaped the Lagos Emergency Operations Centre (EOC) contact tracing network and travelled to Port Harcourt, where he initiated another chain of infections, involving three cases [7,8].

On July 23, 2015, the Nigerian authorities declared an EVD emergency, and activated the EOC to rapidly respond to the outbreak. In collaboration with partner agencies, the government, using existing public health assets, was able to set up six response teams, namely i) point of entry, ii) epidemiology/surveillance, iii) case management/infection control, iv) laboratory services, v) social mobilization, and vi) management/coordination, to rapidly contain the epidemic. When WHO declared Nigeria free of EVD [9] on October 20, 2014, the EOC had identified 894 contacts in three states (Lagos, Rivers, and Enugu), making approximately 18,500 face-to-face contact visits. There were a total of 20 confirmed or probable EVD cases, of whom eight died [7].

Therefore, despite the success of Nigeria in containing the EVD outbreak, the country had committed certain errors which cannot be repeated if future epidemics are to be rapidly contained. In these instances, there was a delay in providing funds for the early and crucial activities of the EOC. Official pre-outbreak information provided to the public was incorrect, creating a false sense of confidence regarding government preparedness for controlling EVD.

## EVD is no stranger to Africa

Since 1976, when EVD was first discovered in Africa, there have been 24 other outbreaks, aside from the current West African outbreak, with a total of 2,900 cases and 1,583 deaths [10]. As of April 15, 2015, the current outbreaks have accounted for around ten times this number: 25,826 cases and 10,704 deaths [4]. Several reasons have been given for the current unprecedented and prolonged EVD outbreak. These factors include, among others, i) the unreliable disease surveillance system which failed to detect and notify the outbreak at onset; ii) an official national and community denial of the occurrence of the epidemic; iii) the fragile and weak heath care delivery system which was incapable of managing the cases and containing the epidemic; iv) cultural practices associated with unsafe burial ceremonies; and v) ease and rapid mass movement of people between rural areas and urban centres, as well as across largely uncontrolled international borders.

Initial international response to the outbreak was described by Médecins Sans Frontières as “*slow and derisory, ..... irresponsible*” [11]. When international response was eventually in place, it was uncoordinated, making the initial global effort seem like a “*consortium of confusion*”.

## Unapproved EVD drugs and vaccines, Africa’s magic bullet, or is there another way?

Having failed to apply standard infection control guidelines to contain the epidemic, frantic efforts were made at global level. The focus temporarily shifted to using available, but unapproved, therapies on compassionate grounds [12]. Therefore, efforts were redoubled to find the ‘magic bullet’, namely drugs and vaccines, for the treatment of cases and to stem the tide of the epidemic. It is becoming clear, with the reduction in number of cases in Liberia and Sierra Leone, that this ‘magic bullet’ to bring the epidemic under control does not consist of drugs or vaccines, but rather a combination of aggressive contact tracing, isolation, and treatment of cases, safe burial ceremonies, and provision of adequate and correct information.

## Capacity building and capacity enablement

Since 1976, when an EVD outbreak was first reported, Africa has battled with no less than 24 episodes of EVD outbreaks [10]. These outbreaks should have provided Africa with opportunities for developing appropriate capacity and enhancing competence needed to deal with any EVD or other disease outbreaks. However, in all the reported EVD outbreaks occurring in Africa for last 38 years, Africa has been at the receiving end of international aid and assistance.

These long years of international aid and assistance for capacity building of Africans in disease control and response, have not actually yielded the desired result on building human resource capacity. This failure to build appropriate capacity is aptly illustrated by an extract from the published diary of a WHO expert, who is assisting with the control of several EVD and other viral hemorrhagic fever outbreaks in Africa. He wrote “*We received the first emails on the Guinea event on Friday 14 March. During the weekend, I was travelling in the DRC for a training course on how to take blood samples in Ebola. It was a 3-day training course*” [13]. It is inconceivable to think, and inconsolable to know, that training in ‘how to take blood samples in Ebola’ is the type of external technical assistance offered to health workers in DRC, a country where 38 years ago, the first EVD case was detected, and which has brought under control at least seven other EVD outbreaks [14]. DRC nationals have provided and continue to provide technical assistance to other African countries on EVD control.

By its nature and character, international assistance or aid, be it for economic development or research collaboration, tends to benefit the donor more than the recipient. A Director of a UK funded research center in Africa, wrote this piece in an annual report of the center: “*Perhaps the most important achievement of the Unit during the past five years has been the training of a group of dedicated scientists and clinicians who have gone on to establish their own highly successful groups elsewhere in the tropics and in the UK and who continue to maintain the high reputation of the UK in tropical medicine*” [15].

This is not an isolated case. A cursory review of the activities of foreign funded research centres in Africa revealed that scientists of the donor countries have benefitted more than their African counterparts in areas of research publication, competition for grants, and international recognition [16]. In many international conferences where issues concerning diseases of African origin and which are endemic in Africa, most, if not all the researchers and paper presenters are non-Africans. The disease outbreaks in Africa have become opportunities for foreign researchers to fine-tune their skills, enabling them to solve African problems, doing for Africa what African scientists should be doing for Africa.

Meanwhile, African scientists remain dependent on the crumbs from the tables of external donors; reduced to mere sample collectors and impotent contributors to disease prevention and control efforts locally or globally. If the next epidemic is not to catch Africa unprepared, this character and nature of external aid and assistance for research collaboration with Africa must change. To reduce dependence on external aid, Africa must reorganize her priorities, and invest more funds in research and research capacity building. African governments must create a conducive environment for African scientists to function with relevance, effectively and productively, freed from the bondage of dependency on foreign external support. On their part, developed countries should ease off on their dominance and control of the processes of global disease surveillance and create an atmosphere for genuine collaboration based on mutual appreciation and respect [17]. Only then can developing countries truly ‘own’ the processes of establishing a sustainable and reliable disease surveillance system for the prevention and control of disease and contribute to global health.

## Conclusions

Even as agencies, foundations, governments, and institutions outside Africa are holding meetings to assess the lessons learnt from the EVD disaster and map out strategies for better response and control of future epidemics, EVD devastated African countries are enmeshed in other battles – misuse of EVD funds [18,19] and the inability to fully utilize aid and resources donated by international agencies. Therefore, is Africa prepared for the next epidemic? Has Africa learnt important lessons from the current EVD outbreak in West Africa? Will Africa still be helpless and totally dependent on international agencies for assistance to control any future epidemic? Will the scientists and governments of the developed world continue to so control the processes of a sustainable global disease surveillance system, leaving no meaningful role for participants from the developing countries? The answers of a pessimist would be no to the first two and yes to the last two questions. For the opposite to occur, Africa must accept that her self-imposed poverty status is not due to a lack of resources, but rather to their misuse, and that it can be reversed. This requires purposeful leadership and rightly guided followership. The African researcher or scientist must evaluate the needs of the society and become relevant to the society through adaptation and development of technologies that positively impact on the daily life of citizens. The poor standard of living and status of life in Africa places an extra burden on the African researcher or scientist that transcends the confines of learning and expertise. The researcher or scientist must take on the extra roles of guide, teacher, mentor, and beacon to society. Therein lies the true relevance of the African scientist or researcher. International aid will not build the capacity for such desired relevance; it must be funded with home-based resources and through governments with the right priorities.
